# Development of a SCAR Marker-Based Diagnostic Method for the Detection of the Citrus Target Spot Pathogen* Pseudofabraea citricarpa*

**DOI:** 10.1155/2018/7128903

**Published:** 2018-06-03

**Authors:** Yuheng Yang, Junhua Hu, Fajing Chen, Dekuan Ding, Changyong Zhou

**Affiliations:** ^1^College of Plant Protection, Southwest University, Chongqing 400715, China; ^2^Institute for Bioscience and Biotechnology Research, University of Maryland, College Park, College Park, MD 20850, USA; ^3^Citrus Research Institute, Southwest University, Chongqing 400712, China; ^4^Citrus Institute of Chenggu County, Shaanxi 723200, China

## Abstract

Target spot, a recently observed citrus disease that is caused by* Pseudofabraea citricarpa*, can cause substantial economic losses in citrus production. In this study, a 797 bp marker specific to* Ps. citricarpa* was identified via random amplified polymorphic DNA (RAPD) technique. The primer pair Pc-SFP/Pc-SRP, which was designed from RAPD amplicons, was utilized as a sequence-characterized amplified region (SCAR) marker. This marker identified* Ps. citricarpa* with a single and distinct band of 389 bp but did not amplify DNA from other tested fungal species. The PCR assay was highly sensitive to the target DNA at picogram levels and could reliably amplify* Ps. citricarpa *sequences with the Pc-SFP/Pc-SRP primer pair. The SCAR marker that was identified in the present study can facilitate rapid decision-making and precise disease forecasting and management.

## 1. Introduction

**Figure 1:**
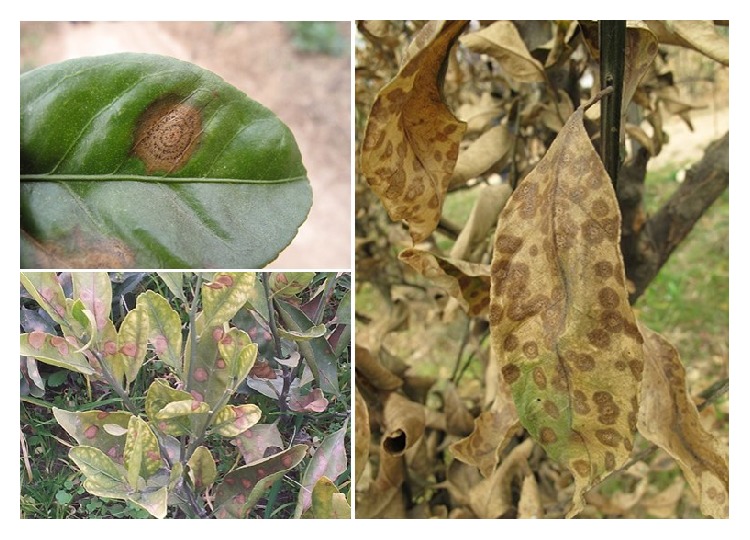
Symptoms of citrus target spot caused by* Pseudofabraea citricarpa*.

Target spot, a new leaf-spotting disease of citrus first described in China, has caused considerable economic losses in local citrus production [1]. The target spot pathogen was identified as* Cryptosporiopsis citricarpa* based on Koch's postulates and morphological and molecular phylogenetic characteristics [1] and then reclassified to the monotypic genus* Pseudofabraea* [2]. This fungal pathogen could infect both Satsuma mandarin* (Citrus unshiu)* and kumquat* (Fortunella margarita)* in orchards [1]. Unlike diseases that usually occur on the young leaves of citrus during warm and humid seasons, target spot occurs during late winter and early spring and causes severe leaf spotting or even defoliation (Figure 1). However, target spot is difficult to diagnose accurately based solely on experience and subjective judgment. Once the disease becomes epidemic, fungicide application was difficult to control effectively. Therefore, monitoring the disease in the citrus orchards plays a key role in effective control of target spot.

Citrus infected by* Ps. citricarpa* does not show any symptoms at early stages of invasion, which is difficult to determine the primary infection potential, and early molecular detection of this pathogen. In recent decades, molecular methods, particularly nucleic acid-based methods, have been applied to identify and detect plant pathogens; these methods can overcome uncertain diagnosis or pathogen taxonomy and enable the rapid and accurate detection and quantification of pathogens [3, 4]. Sequence-characterized amplified region (SCAR), a kind of reliable PCR-based molecular marker, has been developed to detect plant pathogens, such as* Magnaporthe grisea* [5],* Puccinia striiformis* [6], and* Fusarium oxysporum* [7]. The use of the SCAR markers simplifies identification and promotes the development of prevention strategies that are superior to traditional methods.

In the current study, we developed a useful SCAR marker via the simple random amplified polymorphic DNA (RAPD) technique [8, 9] and establish a sensitive and simple PCR-based method for the rapid molecular identification and differentiation of* Ps. citricarpa* from other fungal pathogens of citrus.

## 2. Materials and Methods

### 2.1. Fungal Pathogens

*Ps. citricarpa* strains were isolated from citrus leaves or shoots with disease symptoms. The diseased plant materials were obtained from local orchards. Five fungal pathogens of citrus leaves were collected from Citrus Research Institute, Southwest University. The pathogens included* Alternaria alternata*,* Colletotrichum gloeosporioides*,* Diaporthe citri*,* Botrytis cinerea*, and* Phyllosticta citricarpa*. Three fungal pathogens of citrus fruit were collected from the College of Food Science, Southwest University. The pathogens included* Oospora citri-aurantii*,* Penicillium italicum*, and* Pe. digitatum*. Except for* Ps. citricarpa*, which was cultured at 20°C, all tested strains were cultured at 25°C on potato dextrose agar media until the mycelium covered approximately three-quarters of the plates.

### 2.2. DNA Isolation

Approximately 1 g of fresh fungal mycelium and approximately 0.3 g of field-infected citrus tissues were snap-frozen in liquid nitrogen, and ground to a fine powder with a mortar and pestle. Genomic DNA was extracted via the CTAB method [10]. DNA samples were dissolved in 0.1x TE buffer, quantified, and adjusted to a final concentration of 100 ng/*μ*L for PCR amplification.

### 2.3. RAPD Analysis

RAPD amplification was conducted with 15 *μ*L of reaction mixture with 40 random primers (Table S1). Each reaction tube contained 100 ng of DNA, 1 U of rTaq DNA polymerase (Takara Co., China), 100 *μ*mol/L of each dNTP, 1.5 *μ*L of 10x Taq DNA polymerase buffer with 1.5 mmol/L MgCl_2_, and 1.0 *μ*L of random primer (10 mmol/L). PCR amplification was performed in a DNA thermocycler (Bio-Rad S1000™) with the following conditions: 94°C for 5 min, 35 cycles at 94°C for 30 s, 36°C for 30 s, and 72°C for 90 s with a final extension at 72°C for 10 min. The amplified PCR products were resolved on 1.5% agarose gels, followed by GoldView staining and visualization under UV light.

### 2.4. Amplicon Cloning and Sequencing

The amplicon, which was specific to* Ps. citricarpa* but absent in the other eight species, was identified and purified with a gel extraction mini kit (Tiangen Biotech Co., China). The purified DNA products were cloned into a pGEM-T Easy vector (Promega Co., USA) and introduced into the competent cells of* Escherichia coli* strain DH5*α* in accordance with manufacturer's instructions. Subsequently, the positive clones were sequenced by Shanghai Biotech Co.

### 2.5. Primer Design and Establishment of Detection System

Based on the sequenced RAPD amplicons, the specific SCAR primers (Table 1) Pc-SFP (specific forward primer) and Pc-SRP (specific reverse primer) were designed using Primer Premier 6 software (Premier Biosoft International, USA). A 20 *μ*L reaction system was developed to simplify the detection system. The system contained 10 *μ*L of Premix Taq Version 2.0 plus dye (Takara Co., China), 1.0 *μ*L of forward primer (10 mmol/L), 1.0 *μ*L of reverse primer (10 mmol/L), and 100 ng of genomic DNA. Amplifications were conducted in a DNA thermocycler (Bio-Rad S1000) with the following conditions: 94°C for 5 min, 35 cycles at 94°C for 30 s, 55°C for 30 s, and 72°C for 60 s with a final extension at 72°C for 10 min.

### 2.6. Specificity and Sensitivity of the SCAR Marker

All DNA samples, including those from six foliar pathogens and three postharvest pathogens of citrus, were amplified via PCR with the Pc-SPF and Pc-SPR primers (Table 1) to verify the specificity of the SCAR marker. To test detection sensitivity, 50 ng/*μ*L to 5 fg/*μ*L serial dilutions of the DNA of* Ps. citricarpa* strain were used as the DNA templates for PCR amplification under the above thermocycling conditions.

### 2.7. Validating SCAR Marker in Citrus Tissues Collected from Orchards

To confirm the effectiveness of the primer pairs Pc-SPF and Pc-SPR for detecting* Ps. citricarpa* in the field, the primers were used to amplify DNA samples from symptomatic and asymptomatic citrus tissues that were collected diseased orchards. DNA was extracted from leaves and shoots in accordance with the method described above.* Ps. citricarpa* DNA was used as positive control, and the DNA of healthy citrus leaves obtained from greenhouse were used as negative control. PCR amplification was performed with the primers Pc-SPF and Pc-SPR under the above conditions.

## 3. Results

### 3.1. Screening and Sequencing of RAPD Markers for* Ps. citricarpa*

**Figure 2:**
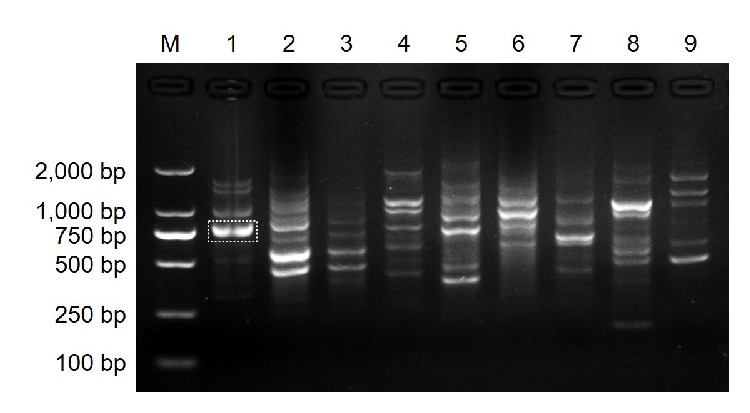
Random amplified polymorphic DNA (RAPD) profiles of* Pseudofabraea citricarpa* and other citrus fungal pathogens obtained with random primer CS38. M, DNA ladder 2000; lane 1,* Ps. citricarpa*; lane 2,* Alternaria alternata*; lane 3,* Colletotrichum gloeosporioides*; lane 4,* Diaporthe citri*; lane 5,* Botrytis cinerea*; lane 6,* Oospora citri-aurantii*; lane 7,* Phyllosticta citricarpa*; lane 8,* Penicillium italicum*; lane 9,* Pe. digitatum*. The dotted box represents the location of the* Ps. citricarpa*-specific band.

**Figure 3:**
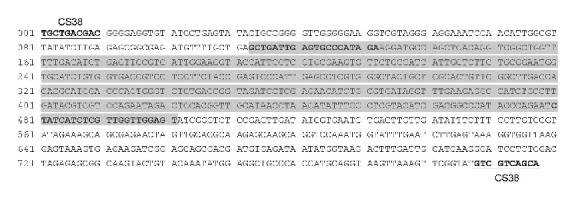
Specific DNA sequence of* Pseudofabraea citricarpa* obtained with the RAPD primer CS38. The gray region indicates the sequence that was amplified by the primer pair Pc-SFP/Pc-SRP (the sequence of the primer pairs were in bold). The first 10 nucleotides of the obtained sequence completely matched the corresponding RAPD primer CS38.

Of the 40 screened RAPD primers, CS38 (5′-TGCTGACGAC-3′) consistently amplified a single intense band of over 750 bp from* Ps. citricarpa*. This band was absent in the eight other pathogens (Figure 2). This differential band was selected to develop a species-specific SCAR marker and subsequently was cloned and sequenced. The sequencing result showed that the length of the specific amplicon was 797 bp with 50% G + C content (*A* = 188, *T* = 212, *C* = 176, and *G* = 221) (Figure 3). BLAST result revealed that no significant similar sequence had been found at different levels.

### 3.2. Specific SCAR Marker Design and Amplification

**Figure 4:**
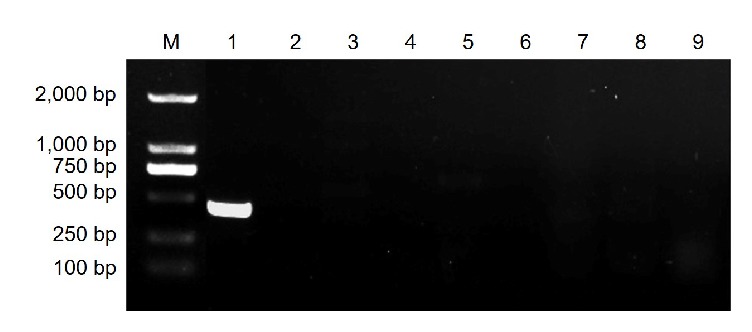
The specificity of PCR product for the detection of* Pseudofabraea citricarpa* using the primer pair Pc-SFP/Pc-SRP. M, DNA ladder 2000; lane 1,* Ps. citricarpa*; lane 2,* Alternaria alternata*; lane 3,* Colletotrichum gloeosporioides*; lane 4,* Diaporthe citri*; lane 5,* Botrytis cinerea*; lane 6,* Oospora citri-aurantii*; lane 7,* Phyllosticta citricarpa*; lane 8,* Penicillium italicum*; lane 9,* Pe. digitatum*.

The primer pair Pc-SFP/Pc-SRP (Table 1) was designed using Primer Premier 6.0 software (Premier Biosoft International) based on the sequence of the specific amplicon. When Pc-SFP and Pc-SRP were used to amplify genomic DNA from the nine selected pathogens, a single and distinct band of 389 bp was only observed in* Ps. citricarpa* (Figure 4). Sequencing analysis showed the amplicon was the expected* Ps. citricarpa* fragment, indicating that the designed SCAR marker is specific for the citrus target spot pathogen.

### 3.3. Sensitivity Test of the Detection System

**Figure 5:**
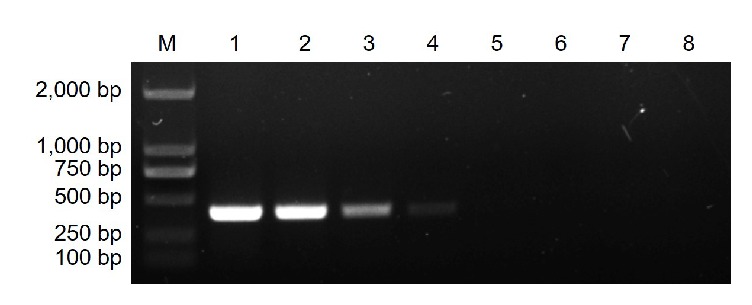
The PCR sensitivity of the primer pair Pc-SFP/Pc-SRP with a serial dilution of* Pseudofabraea citricarpa* DNA. M, DNA ladder 2000; lane 1, 50 ng/*μ*L; lane 2, 5 ng/*μ*L; lane 3, 500 pg/*μ*L; lane 4, 50 pg/*μ*L; lane 5, 5 pg/*μ*L; lane 6, 500 fg/*μ*L; lane 7, 50 fg/*μ*L; lane 8, 5 fg/*μ*L.

To test the sensitivity of the specific marker for detecting* Ps. citricarpa,* serial dilutions of* Ps. citricarpa* DNA were used as templates in the PCR assay with Pc-SFP and Pc-SRP primers. The results revealed that the SCAR marker could detect* Ps. citricarpa* DNA at levels as low as 50 pg/*μ*L (Figure 5).

### 3.4. Detection of* Ps. citricarpa* in Orchards

**Figure 6:**
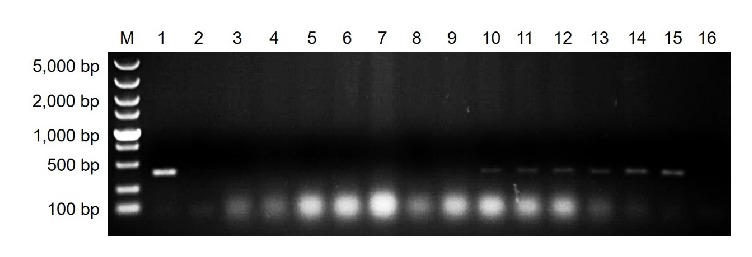
PCR amplification using DNA extracted from citrus samples that were collected from orchards with target spot. M, DNA ladder 5000; lane 1, positive control (*Ps. citricarpa* DNA); lanes 2–15, citrus leaves or shoots without any visible symptoms; lane 16, negative control (uninfected citrus leaf DNA).

To test the reliability of the* Ps. citricarpa*-specific SCAR marker Pc-SFP and Pc-SRP, citrus leaves and shoots without any visible symptoms were collected from diseased orchards and were used for the verification test. The expected 389 bp bands were obtained from portions of the selected samples (Figure 6). No PCR product was amplified in the negative control (uninfected citrus leaves). The results validated the reliability of the designed SCAR marker.

## 4. Discussion

Given that knowledge on the infection cycle and disease epidemics of citrus target spot is limited, the disease has been mistaken as a brown spot or anthracnose for prevention and control for a long time, which caused poor control effects [11]. The sensitivity tests showed that the SCAR marker could detect as low as 50 pg/*μ*L of* Ps. citricarpa* DNA extracted from mycelia and from citrus leaves or shoots collected diseased orchards, but not from healthy leaves (Figure 5). These results indicated that the proposed amplification system could help illustrate the oversummering mechanism and occurrence characteristics of citrus target spot, which will be useful for the effective forecasting and management of this disease.

RAPD analysis reveals a high degree of polymorphism even without the DNA sequence information of the species; moreover, RAPD is easy to perform [12]. Given the advantages of low workload, rapidity, and high efficiency compared with traditional identification methods, RAPD-based SCAR markers are extensively used for the in planta detection of several plant pathogens [5, 13, 14]. The SCAR marker developed in this study can also facilitate rapid decision-making and precise early season disease management to reduce the risk of* Ps. citricarpa* epidemics.

## Figures and Tables

**Table 1:** *Pseudofabraea citricarpa*-specific SCAR primers designed from sequenced RAPD amplicons.

RAPD primer	SCAR marker	Number of base pairs (bp)	Nucleotide sequence	G + C content (%)	Annealing temperature
CS38	Pc-SFP	20	5′-GCTGATTGAGTGCCCATAGA-3′	50	55°C
	Pc-SRP	22	5′-ACTCCAACCAACGAGATGATAG-3′	45	

## Supplementary Materials

Supplementary Materials Table S1: sequence of random amplified polymorphic DNA (RAPD) PCR primers used in this study.
