# Supplementary material for: Development of a SCAR Marker-Based Diagnostic Method for the Detection of the Citrus Target Spot Pathogen Pseudofabraea citricarpa
Source: Biomed Res Int. 2018 Jun 3;2018:7128903. doi: 10.1155/2018/7128903 (PMC6008725; doi:10.1155/2018/7128903)
Supplement: Supplementary Materials — Table S1: sequence of random amplified polymorphic DNA (RAPD) PCR primers used in this study. [file 7128903.f1.docx]

**Table S1.** Sequence of Random amplified polymorphic DNA (RAPD)

PCR primers used in this study.

| Primer code | Nucleotide sequence | Primer code | Nucleotide sequence |
| --- | --- | --- | --- |
| CS1 | 5’-CGCCGAGGAA-3’ | CS21 | 5’-GGACTGGAGT-3’ |
| CS2 | 5’-GCTGTTTCCT-3’ | CS22 | 5’-GTCCACACGG-3’ |
| CS3 | 5’-TGAATCGGCC-3’ | CS23 | 5’-CCACAGCAGT-3’ |
| CS4 | 5’-TTGTCTTCGG-3’ | CS24 | 5’-TGCCGAGCTG-3’ |
| CS5 | 5’-TCCGAAATCA-3’ | CS25 | 5’-CTCTGGAGAC-3’ |
| CS6 | 5’-TCCCGGCCGC-3’ | CS26 | 5’-CCTACGTCAG-3’ |
| CS7 | 5’-GTCGTTCTGA-3’ | CS27 | 5’-TGCCGAGCTG-3’ |
| CS8 | 5’-TTTGCGTATT-3’ | CS28 | 5’-TCACCACGGT-3’ |
| CS9 | 5’-ATTGCAACTG-3’ | CS29 | 5’-CTACTGCCGT-3’ |
| CS10 | 5’-TGATCCTTCC-3’ | CS30 | 5’-TTTGGGGCCT-3’ |
| CS11 | 5’-GGCATGCCTG-3’ | CS31 | 5’-CTGGCGAACT-3’ |
| CS12 | 5’-GCGGCGCCGT-3’ | CS32 | 5’-CCTGCTCATC-3’ |
| CS13 | 5’-CACATTGCGC-3’ | CS33 | 5’-GGGCCAATGT-3’ |
| CS14 | 5’-GGTGAACCTG-3’ | CS34 | 5’-TGCCGGCTTG-3’ |
| CS15 | 5’-GGGCCTCGCC-3’ | CS35 | 5’-GGTGAGGTCA-3’ |
| CS16 | 5’-CGGAAGGATC-3’ | CS36 | 5’-GGCTAACCGA-3’ |
| CS17 | 5’-CCGCCGAAGG-3’ | CS37 | 5’-CTACCAGGGA-3’ |
| CS18 | 5’-GAACGCAGCG-3’ | CS38 | 5’-TGCTGACGAC-3’ |
| CS19 | 5’-CGATAAGTAA-3’ | CS39 | 5’-TCGGTGAGTC-3’ |
| CS20 | 5’-CCAGCTTGGT-3’ | CS40 | 5’-GGAGCAGCAA-3’ |
